# Application of Twitter and web news mining in infectious disease surveillance systems and prospects for public health

**DOI:** 10.3205/dgkh000334

**Published:** 2019-12-02

**Authors:** Kia Jahanbin, Fereshte Rahmanian, Vahid Rahmanian, Abdolreza Sotoodeh Jahromi

**Affiliations:** 1 Research Center for social determinants of health, Jahrom University of Medical Sciences, Jahrom, Iran; 2 Zoonoses Research Center, Jahrom University of Medical Sciences, Jahrom, Iran

**Keywords:** fuzzy classification, surveillance system, Twitter, text mining, infectious disease

## Abstract

**Aims:** With the advancements of communication technology and growing access to social networks, these networks now play an important role in the dissemination of information and news without going through the time-consuming channels of official news networks. Analysis of social networking data is a new, interesting branch of text mining science. This study aimed to develop a text mining technique for extracting information about infectious diseases from tweets and news on social media.

**Methods:** A method called “Fuzzy Algorithm for Extraction, Monitoring, and Classification of Infectious Diseases” (FAEMC-ID) was developed by the use of fuzzy modeling of the Takagi-Sugeno-Kang type. In addition to the real-time classification, the method is able to update its vocabulary for new keywords and visualize the classified data on the world map to mark the high risk areas.

**Results:** As an example, the monitoring was performed for measles-related news items over a 183-hour period from 01/03/2019 (01:00 am) to 08/03/2019 (12:00 pm), which were related to 2,870 tweets from 2,556 users. This monitoring showed that the number of tweets posted from each region ranged from 1 to 47, with the highest number, 47 tweets, belonging to Canada. The origins of most measles-related news were in the Americas and Europe, and they were mostly from the United States and Canada.

**Conclusion:** The performance analysis of the developed method in comparison with other algorithms in the literature demonstrated the excellent precision of the method with a recall ratio of 88.41% and the high inter-correlation of data in each class. The proposed algorithm can also be used in the development of more effective monitoring and tracking systems for other human and even animal health hazards.

## Zusammenfassung

**Zielsetzung:** Mit der Weiterentwicklung der Kommunikationstechnologie und dem wachsenden Zugang zu sozialen Netzwerken spielen diese Netzwerke eine wichtige Rolle zur Verbreitung von Informationen und Nachrichten, ohne dass die zeitaufwendigen Kanäle offizieller Nachrichtennetzwerke durchlaufen werden müssen. Die Analyse sozialer Netzwerkdaten ist ein neuer, interessanter Zweig der Text-Mining-Wissenschaft. Diese Studie zielt darauf ab, eine Text-Mining-Technik zu entwickeln, um Informationen über Infektionskrankheiten aus Tweets und Nachrichten in sozialen Medien zu extrahieren.

**Methode:** Als Analysemethode wurde der sog. „Fuzzy-Algorithmus zur Extraktion, Überwachung und Klassifizierung von Infektionskrankheiten“ (FAEMC-ID) unter Verwendung des Fuzzy-Modells des Takagi-Sugeno-Kang-Typs entwickelt. Zusätzlich zur Echtzeitklassifizierung kann die Methode neue Schlüsselwörter aktualisieren und die klassifizierten Daten auf der Weltkarte visualisieren, um Hochrisikobereiche zu markieren.

**Ergebnisse:** Als Beispiel wurde das Monitoring für Nachrichten mit Bezug zu Masern über einen Zeitraum von 183 Stunden vom 01.03.2014 (01:00 Uhr) bis 08.03.2014 (12:00 Uhr) durchgeführt, das 2.870 Tweets von 2.556 Benutzern umfasste. Das Monitoring ergab als Anzahl der von jeder Region geposteten Tweets 1 und 47 mit der höchsten Anzahl von 47 Tweets aus Kanada. Der Ursprung der meisten Nachrichten über Masern war in Amerika und Europa; die Tweets stammten größtenteils aus den Vereinigten Staaten und Kanada.

**Schlussfolgerung:** Die Analyse der entwickelten Methode liefert im Vergleich zu anderen Algorithmen in der Literatur eine ausgezeichnete Präzision mit einer Rückrufquote von 88,41% und einer hohen Interkorrelation der Daten in jeder Klasse. Der vorgeschlagene Algorithmus kann auch zur Entwicklung wirksamer Überwachungs- und Nachverfolgungssysteme für andere Gesundheitsgefahren für Mensch und Tier verwendet werden.

## Introduction

Today, social media generate vast amounts of data on a daily basis in a wide variety of areas including technology, medicine, history, political and social news, sports, and many other fields. These data can be refined and analyzed to extract economically and scientifically valuable knowledge and have therefore piqued the interest of researchers in many areas [1], [2], [3].

In recent years, big data science has emerged as a powerful tool for collecting, storing, managing, and analyzing data on a large scale [4]. Big data can be characterized by five features: volume, variety, velocity, variability, and veracity. Among these features, the most important is the volume or size, according to which data can be classified into three categories [5]:

1. Structured: Data that is organized in a predefined schema.
2. Semi-structured: Data that does not require a predefined schema.
3. Unstructured: Data that is stored without any defined structure or schema.

A great portion of all data produced and consumed across the world is in textual form. The science of text mining is focused on the extraction of high-quality information from textual data [6]. The major applications of text mining include texts categorization, concept/entity extraction, text clustering, text summarization, sentiment analysis, and entity relation modeling [7].

Web-news mining from media and social networks is one of the major applications of text mining in social sciences. An automated news-mining-based system can monitor, analyze, and classify news according to its contents, which is useful not only for managing news articles but also for developing recommenders and security systems [1].

Twitter is one of the world’s most popular social networks. The highly interesting applications of this micro-blogging platform have attracted the attention of researchers. At present, Twitter has over 11 million active users, who post about 6 million tweets every day, including instant messages and comments. Given the easily accessible and extremely rich information contained in tweets, they can be used in a wide range of applications, including the analysis of political trends, product performance, and the monitoring of health-related events [8], [9] .

In the model proposed in this paper, the unstructured data about infectious diseases like influenza, HIV/AIDS, malaria, measles, poliomyelitis, tuberculosis, plague, Ebola and cholera are extracted from Twitter and then subjected to text cleanup, term filtering, and finally categorization operations. Since the focus of the work is on real-time application, the model is implemented with the help of a fuzzy rule-based evolutionary algorithm called Eclass1-MIMO.

### Literature review

In 2014, the term “social big data” was used for the first time to refer to the data generated by social networks [4], [10]. This includes, for example, the 30 million tweets posted every day, the 3,000 photos uploaded to Flickr every minute, and the 15 million blog posts written on a daily basis. These social networking data can have scientifically and economically significant uses in many fields including sociology, psychology, politics, commerce, and healthcare [8], [11], [12], [13].

Text mining can be discussed from two perspectives – the type of knowledge extracted and applications. Applications of text mining can be categorized as follows:

- **Security applications: **Text mining packages have extensive use in security software, especially for analyzing online plain texts such as websites and weblogs for national security protection purposes [8].
- **Biomedical applications: **A wide range of text mining tools and software has been developed for biomedical applications [10]. For example, PubGene is a well-known Internet service that combines biomedical text mining with network visualization [14].
- **Online media applications: **Media corporations such as the Tribune Company have utilized text mining to achieve enhanced data clarity and create more interesting contents for readers. This science has also been used in the public sector to develop software for the monitoring and tracking of terrorist activities [15].
- **Business and marketing applications:** Text mining is finding extensive use in business and marketing intelligence and particularly in customer relations management [16], [17].
- **Sentiment analysis: **Sentiment analysis can be discussed from the perspective of the type of information extracted and its application. For example, sentiment analysis has been used for the analysis of movie reviews [18] and also for comment recognition in the field of artificial emotional intelligence [2], [19].
- **Academic applications: **Text mining is one of the major tools that large publishers use for data categorization and retrieval from large databases [8].
- **Text categorization: **Text categorization is an automatic process whereby text data are organized into multiple predefined categories or classes. One of the applications of text categorization is the opinion categorization, which gives an insight into the opinion of users of social networks like Facebook or Twitter about a certain topic (e.g. a law, a treatment, a political view, etc.) [20].
- **Text clustering: **Unlike text categorization, text clustering is focused on the unsupervised management of text documents [21].
- **Text summarization:** Automatic text summarization algorithms are language-independent (multilingual) tools for generating a summary of a text [5], [22], [23].

This paper presents a method based on a Takagi-Sugeno-Kang (TSK) fuzzy system called the Eclass1-MIMO model for the categorization of news on Twitter about infectious diseases with epidemic potential. In developing the method, the authors aim to create an accurate text categorization system with real-time applicability for marking high risk areas based on tweets for improved monitoring and timely control of growing epidemics and related damage.

## Methods

One of the most effective ways to prevent and control epidemics is to monitor and track the news about the spread of contagious diseases. This section explains the general frame and main structure of the proposed model for the collection of raw data about a select group of contagious diseases from related news and tweets and the analysis of these data. 

The proposed method consists of 4 phases:

1. Data cleanup and integration and term extraction
2. Web and tweet crawling
3. Applying fuzzy rules and fuzzy classifier
4. Visualization

The first phase consists of data cleanup, data integration, and term extraction steps. The term extraction step consists of letter case homogenization (transforming all words to lowercase), tokenization, stemming, filtering stop words (removing pronouns, auxiliary verbs, and so on), and term filtering with the TF-IDF method.

In the proposed method, classification and evolving fuzzy rules are developed with the help of fuzzy rule-based classification package (FRBS) [24], [25]. The evolving fuzzy system plays a fundamental role in the text analysis, i.e. updating the terms being extracted from the database [26]. This is important because, considering the large volume and unpredictable nature of the news and tweets related to infectious diseases and the likely emergence of new terms over time, the terms used in classification must be regularly updated. To resolve this issue, the proposed method makes use of evolving fuzzy rules and implements the text classification scheme with the Eclass1-MIMO method based on TSK rules [27], [28], [29], [30].

**Figure 1:**
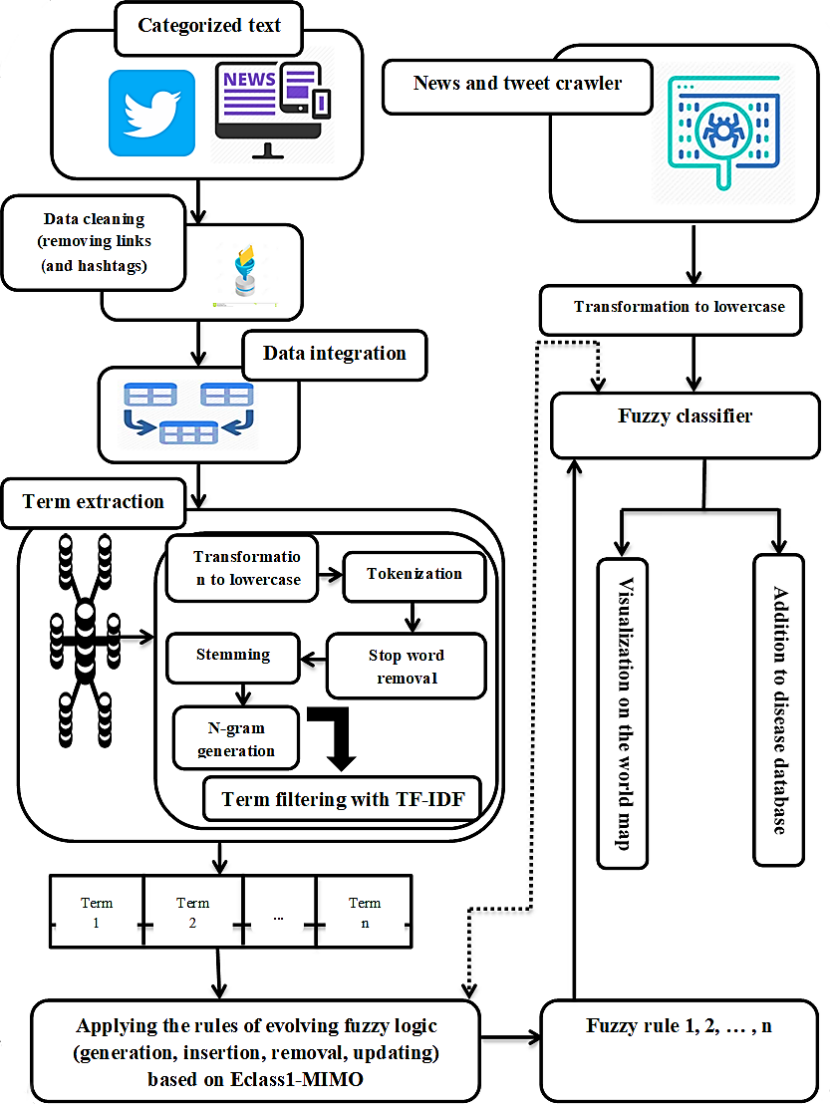
Framework of data collection and monitoring for infectious diseases

The visualization component of the proposed method aims to assist real-time monitoring and tracking of the onset and spread of epidemics, which can greatly contribute to the efficacy of active health and research systems in this area. Details of the proposed method are illustrated in Figure 1 (Fig. 1).

### Data cleanup, data integration, and term extraction

As shown in Figure 1 (Fig. 1), the first phase of the proposed method consists of three steps:

1. Text cleanup: This step involves processing the tweet and news contents to remove redundant characters such as @ (“at” sign), # (hashtag), rt (for retweets), emotions, metadata, links, etc., which should be cleaned before classification [31]
2. Data integration: After text cleanup, tweets and news are integrated into related classifications.
3. Term extraction: This step consists of the following processes:

**- Tokenization:** In this step, the streams of textual data are decomposed into words, symbols, phrases, and other meaningful elements as well as keywords that are valuable for classification, clustering and analysis of texts [1], [31].

**- Homogenization:** In this step, all words in the database are transformed to lowercase in order to prevent redundant terms [31].

**- Stopword filtering:** This step involves finding and removing pronouns, prepositions, and “to be” verbs from the text [32].

**- Stemming:** In this step, the inflected and derived words (with prefixes, suffixes, etc.) are converted to their base form in order to reduce the number of redundant terms [1], [31]. In this work, stemming is done with the help of the Snowball algorithm [32].

**- n-gram generation:** n-gram is an alternating sequence of n items (characters, letters, etc.); an n-gram is said to be a unigram if n=1, bigram if n=2, and trigram if n=3. n-gram generation has extensive use in language identification [20] and speech recognition, and contributes to the identification of keywords that are not valuable by themselves. In this study, the learning accuracy of the model is improved by the use of bigrams [33], [34].

**- Term filtering:** The tokenization step extracts all terms of each tweet without considering the frequency of each term, which can reflect its importance. The term filtering step involves removing the terms that rarely appear in the text, the terms that have a constant distribution, and the terms that appear too frequently in the text in order to prevent the redundant growth of the term set [1].

### Database collection method

In the proposed method, the news about smallpox, influenza, malaria, measles, poliomyelitis, tuberculosis, plague, Ebola and cholera in various news sites is collected by a powerful API called Newsapi. This API collects the news of 54 countries from 134 major news organizations including CNN, BBC, CBC, Washington Post, etc. The code written in Ruby for extracting news about measles, for example from Twitter between 01/03/2019 and 08/03/2019 is presented below:

Require 'open-url'

url='https:/newsapi.org/v2/everything'

‘language=en&’

‘q=measles disease&’

‘from=2019-03-01&’

‘to=2019-03-08&’

‘sortBy=relevancy&’

‘apikey=[write your api]’

Req=open(url)

Response_body-req.read

Put response_body

Tweets crawler was coded with the R language. For example, the following code was used to crawl the HIV-related tweets from 01/03/2019 to 08/03/2019:

Library(twitterR)

Consumer_key=“[your consumer_key]”

Consumer_secret=“[your consumer_key]”

Access_token=“[your access_token]”

Access-secret=“[your access_secret]”

Setup_twitter_oauth(Consumer_key, Consumer_secret, Access_token, Access-secret)

Tw=SearchTwitter(“#HIV”,n=1e4,scince='2019-03-01')

### Application of fuzzy rules and fuzzy classifier

The next step after extracting the terms related to each class involves the application of fuzzy rules and fuzzy classifier. The system developed for this phase consists of two steps:

1. Generation and updating of fuzzy rules
2. Classification of news/tweet related data

In the proposed system, fuzzy rules are generated and updated by a fuzzy model called Eclass1-MIMO, which is a multi-input-multi-output framework based on the rules of the TSK fuzzy system [26]. In addition to using the TSK fuzzy system, the Eclass1-MIMO model can remove useless potential terms with the help of an “aging” mechanism. Using this mechanism, the potential terms that have not been recently used to classify any text are removed from the list of keywords.

The rules of the TSK-based fuzzy model are defined as follows:

1. Rule_i_ = IF (A_1_ is around Port_1_) AND …
  AND (A_n_ is around Prot_n_) Then = J_i_ = A^-t^ *Θ

Where i is the rule number, n is the number of input variables (or terms) in Rule_i_, Port_i_ is the value of variables at A_i_ (obtained using tf-idf), Ā is the vector of input features, i.e. Ā =[1,x_1_,x_2_,…,x_n_ ], and y_i_ is the resulting output. The normalized output is obtained using the following equation [2]:

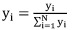

The y_i_ values should sum up to 1: 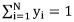


The normalized output can be interpreted to find a match with the existing classes. If classes are binary, “1” means the output is a member of the class, and “0” means it is not. If the objective function has more than two classes or multiple inputs and multiple outputs with (n+1)*k members (where k is the number of classes or classifications, and n is the number of terms), then:

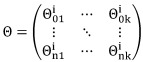

The output of the fuzzy rules related to the k^th^ row of this vector is the normalized output for the class:

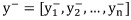

In this study, the choice of using Eclass1-MIMO in the classification algorithm is made because of the dynamic adaptability of fuzzy rules to the changes in the input data stream.

### Fuzzy rule generation, removal, and updating

Provided that the aging condition is met, the fuzzy rules for assigning the news or tweet A_z_ to classes or categories C_j_ are updated in the following steps:

1. Compute the potential terms of news or tweet A_z_
2. Update the patterns (list of all existing terms) according to the potential terms of news or tweet A_z_
3. Insert A_z_ as a new pattern (new pattern of the class C_j_) if necessary.
4. Remove duplicate patterns if necessary

### Comparison of the proposed method with other algorithms

For performance evaluation, the proposed method was compared with the conventional algorithms listed below. This comparison was made in terms of accuracy, misclassification, Kappa statistic, and absolute error.

1. Naïve Bayes algorithm: This is a simple classifier based on the Bayes theory, which has no configurable parameters [35].
2. Bayesian network algorithm: Bayesian networks (BNs) are a family of probabilistic graphic models (GMs) developed by the combination of graph theory, probability theory, and statistics. In this algorithm, each vertex of the graph represents a random variable and the edges between vertices represent the probabilistic dependence between the corresponding random variables [36].
3. Deep learning: Deep learning is a multi-layer feed-forward artificial neural network that is trained by a stochastic gradient descent scheme using back-propagation [37].
4. K-nearest neighbor’s algorithm (KNN): In this algorithm, the parameter K is the number of closest training examples or the number of nearest neighbors in the feature space. After receiving K as an input, this puts the K nearest neighbors of an object to the same class. In this algorithm, distance is measured based on a distance criterion like Euclidian distance [38].
5. Learning Vector Quantization (LVQ) neural network: LVQ neural network is an artificial neural network based on local competitive learning. In this network, neurons are called codebooks or prototypes [39].
6. Support Vector Machine (SVM): SVM algorithms are supervised/unsupervised learning models developed for classification and regression analysis [40].

The comparison between the classification methods was performed by the use two of metrics, accuracy and confusion matrix, which represent, respectively, the degree and extent of text classification precision.

## Results

The collected database consisted of 10,000 news items and tweets (selected), which, after data cleanup and integration, yielded 1,100 keywords in 9 classes. After applying a pruning technique (in the 10%–30% range) to remove the terms with low tf-idf index, the results provided in Table 1 (Tab. 1) were obtained. It should be noted that to improve the accuracy and speed of the process in real-time applications, pruning was performed with p<20%.

**Figure 2:**
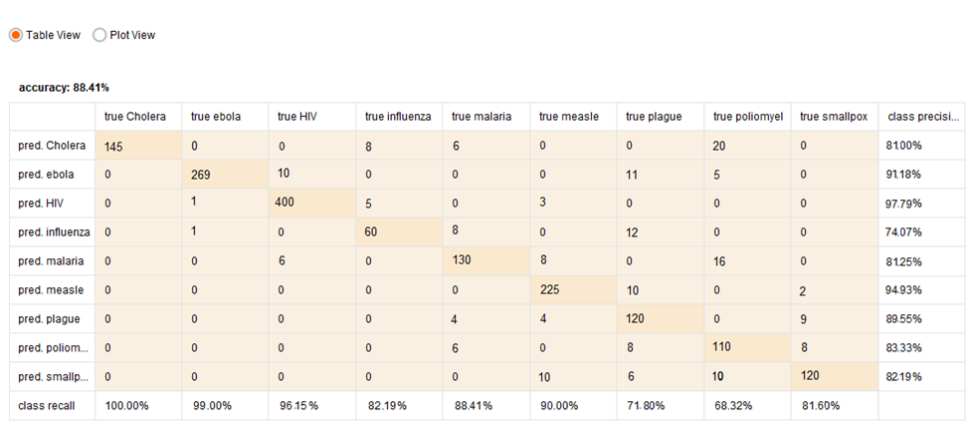
Accuracy and confusion matrix of the proposed algorithm

Figure 2 (Fig. 2) shows the accuracy and confusion matrix of the proposed algorithm, named Fuzzy Algorithm for Extraction, Monitoring and Classification of infectious Diseases or FAEMC-ID. While using FAEMC-ID, the highest and lowest precisions or recall ratios were obtained for cholera and plague. 

As shown in Figure 2 (Fig. 2), unlike in other works [1], the precision of the method increases with the sampling volume. This reflects the applicability of the method to large-scale databases and hence in real-world applications.

In Table 2 (Tab. 2), FAEMC-ID is compared with the conventional algorithms commonly used previous works. This comparison is in terms of accuracy, misclassification, Kappa coefficient, and absolute error. As can be seen, the proposed method exhibits a higher accuracy in the classification of the test data. In addition, the high correlation of data in each class is reflected in the obtained Kappa coefficient.

With an automatic system for extraction of news and comments, one can rapidly build a large database of disease-related events. With the provided visualization process, it is also possible to track the geographical location of the sources of news or comments.

**Figure 3:**
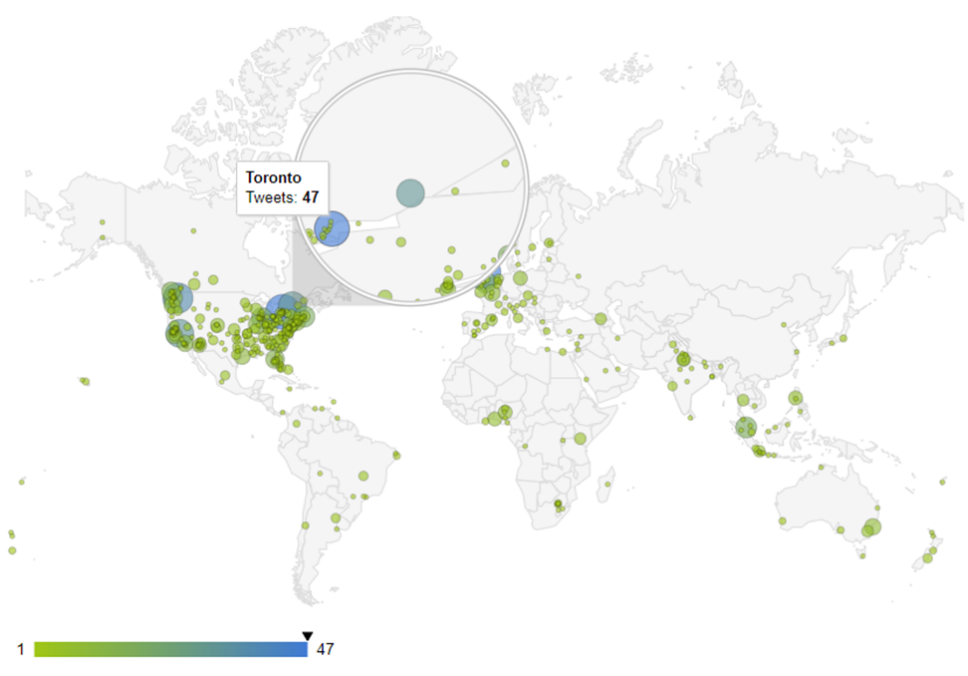
Monitoring of geographical distribution of the tweets about measles from 01:00 am 01/03/2019 to 12:00 pm 08/03/2019

Figure 3 (Fig. 3) shows the results obtained by monitoring measles-related news in a continuous 183-hour period from 01/03/2019 (01:00 am) to 08/03/2019 (12:00 pm), which are related to 2,870 tweets from 2,556 users. The number of tweets posted from each region range from 1 to 47, with the highest number (47 tweets) from Canada. The origins of most measles-related news were in the Americas and Europe, and they were mostly from the United States and Canada. 

**Figure 4:**
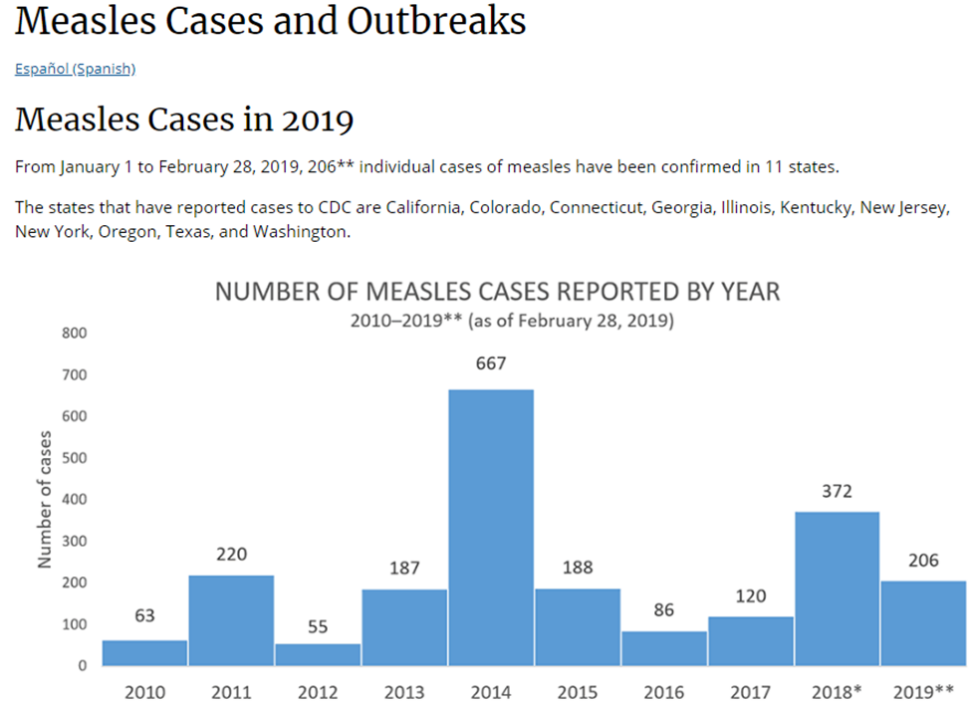
CDC report about the incident of measles in the United States [51]

This is consistent with the map illustrated in Figure 4 (Fig. 4), which was obtained from the United States Centers for Disease Control and Prevention (CDC).

**Figure 5:**
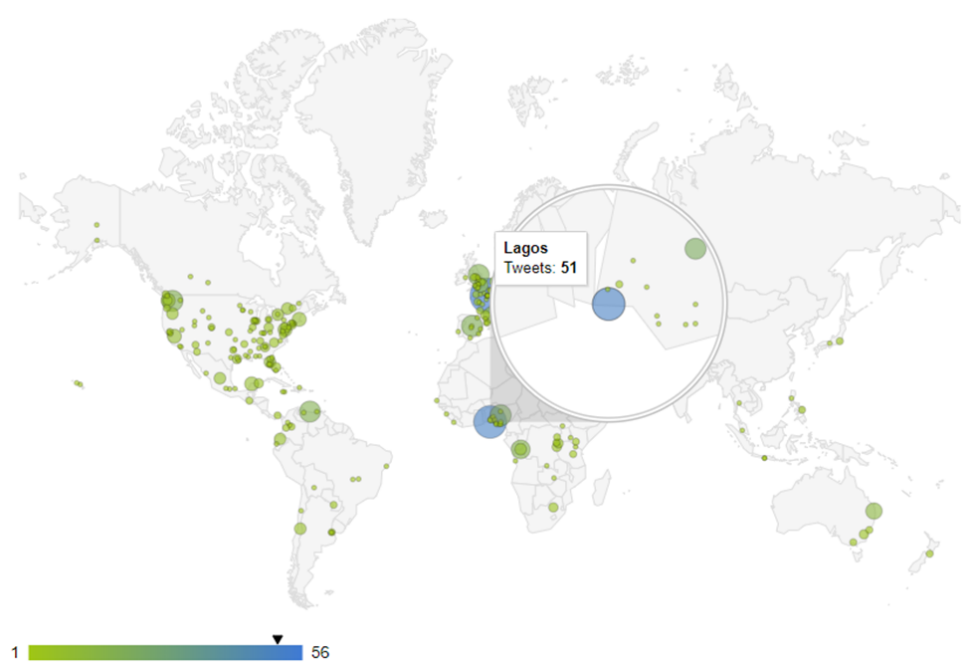
Monitoring of geographical distribution of the tweets about Ebola from 01:00 am 01/03/2019 to 12:00 pm 08/03/2019

**Figure 6:**
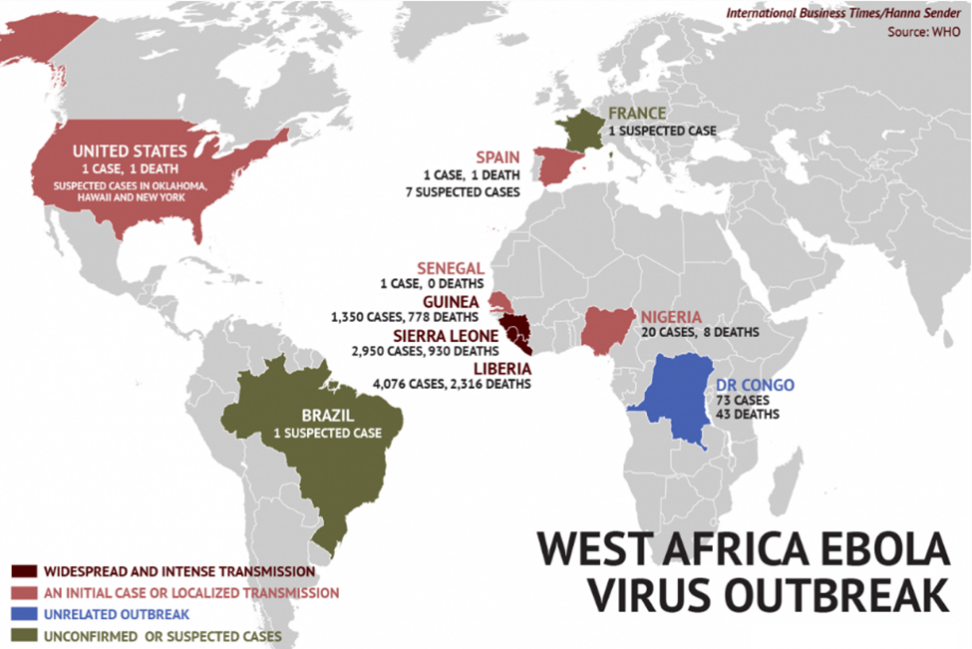
WHO report about the incident of Ebola [52]

**Figure 7:**
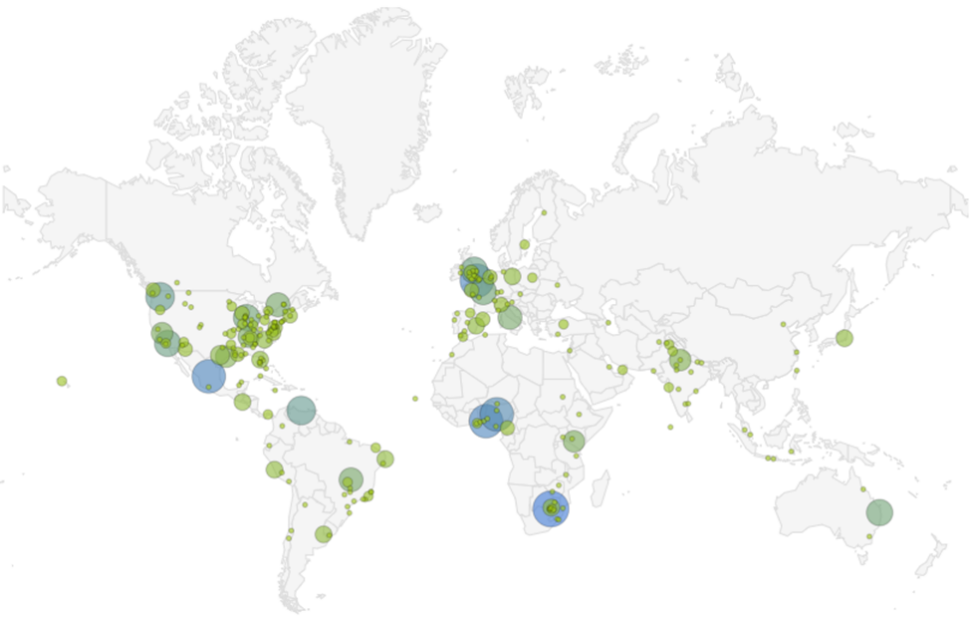
Monitoring of geographical distribution of the tweets about HIV from 01:00 am 01/03/2019 to 12:00 pm 08/03/2019

Figure 5 (Fig. 5) displays the map of Ebola-related news and tweets obtained using the proposed method, and Figure 6 (Fig. 6) shows the map of Ebola epidemics according to the WHO. As can be seen, there is a high degree of consistency between these maps. The map of HIV/AIDS-related news and tweets for the study period is shown in Figure 7 (Fig. 7).

## Discussion

In this study, we developed a new method based on the evolving fuzzy algorithm of TSK type for the extraction, monitoring, storage and visualization of news and tweets about various infectious diseases. To implement the method, more than 10,000 tweets and news were cleaned, integrated and classified with the help of the Eclass1-MIMO method, then visualized on the world map in real-time. 

In recent years, many researchers have worked on classification, clustering, sentiment analysis, opinion mining and development of recommenders based on social data, but most of these works have focused either on news websites or Twitter [1], [41], [42], [43].

The findings of the present study are consistent with those of Angelov PP and Zhou X [26] and Bhattacharyya et al. [25], who reported the high efficacy of evolving fuzzy algorithms in real-time applications in terms of ensuring satisfactory precision, speed, and flexibility.

In the study by Iglesias et al., an evolving fuzzy algorithm with the Eclass1-MIMO method was used to classify the public news into 6 categories of science, health, technology, sports, arts, and commerce [1]. But unlike this model, in the proposed method, increasing the data size not only does not reduce the accuracy but actually improves it. Another advantage of the proposed method over similar works [44], [45], [46], [47] is the ability to visualize the results for improved monitoring and tracking of epidemics.

Also, the geographic origins of tweets posted about measles and Ebola were found to be consistent with official CDC and WHO reports about their incidence during the studied period. This reflects the efficacy of the proposed method in monitoring and tracking the targeted diseases.

The evolving fuzzy method has also been used by Del Jesus [48] to enhance low-grade classification algorithms, by Lughofer [49] to solve the problems of online multiclass classification, and Lughofer [50] for online incremental feature dimension reduction. Our findings about the use of evolving fuzzy method are in agreement with the results of these studies in terms of high accuracy, high correlation of data in each class (kappa coefficient), and efficacy in online multiclass data analysis.

### Study limitation

A limitation of the suggested method is that it cannot be used to monitor and track infectious diseases in areas with poor or no access to social networks such as Twitter and Facebook, and this includes poor countries, where morbidity and mortality due to infectious diseases are noticeably higher.

## Conclusions

This paper presented a method for extraction, monitoring, storage, and visualization of data related to certain infectious diseases through news mining and tweet crawling. The proposed framework consists of four phases, including data collection with a code written in the R-programming language, text cleanup, classification with the evolving fuzzy model Eclass1-MIMO, and visualization. The fuzzy classification component was developed based on fuzzy TSK rules and evolving fuzzy model, and hence is able to update its vocabulary and remain efficient and accurate upon encountering new terms. Moreover, unlike previous methods, the proposed method exhibits satisfactory flexibility regarding the size of input data and can handle large datasets without a decline in classification accuracy. Other notable features of this method include the simultaneous extraction of news from tweets and websites, the real-time classification capability, data storage in one database, and visualization of data in real-time. The analysis of this proposed method in comparison with other algorithms in the literature showed its high accuracy (88.41%) and the high correlation of data within each class. The proposed algorithm can also be used in the development of more effective monitoring and tracking systems for other human and even animal health hazards.

## Notes

### Acknowledgments

We would like to express our gratitude to Dr. Antonio Iglesias at the University of Madrid for the helpful comments, the instructors of the online course “Machine Learning for Data Science and Analytics” provided by Columbia University for giving us better insight into the area of data and text mining, and also the members of the Iran Data Mining Group, who patiently answered our questions.

### Competing interests

The authors declare that they have no competing interests.

## Figures and Tables

**Table 1:**
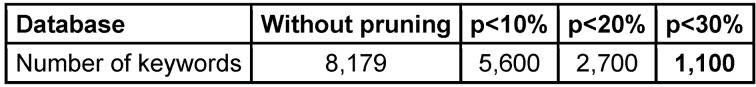
The number of keywords after the application of pruning technique

**Table 2:**
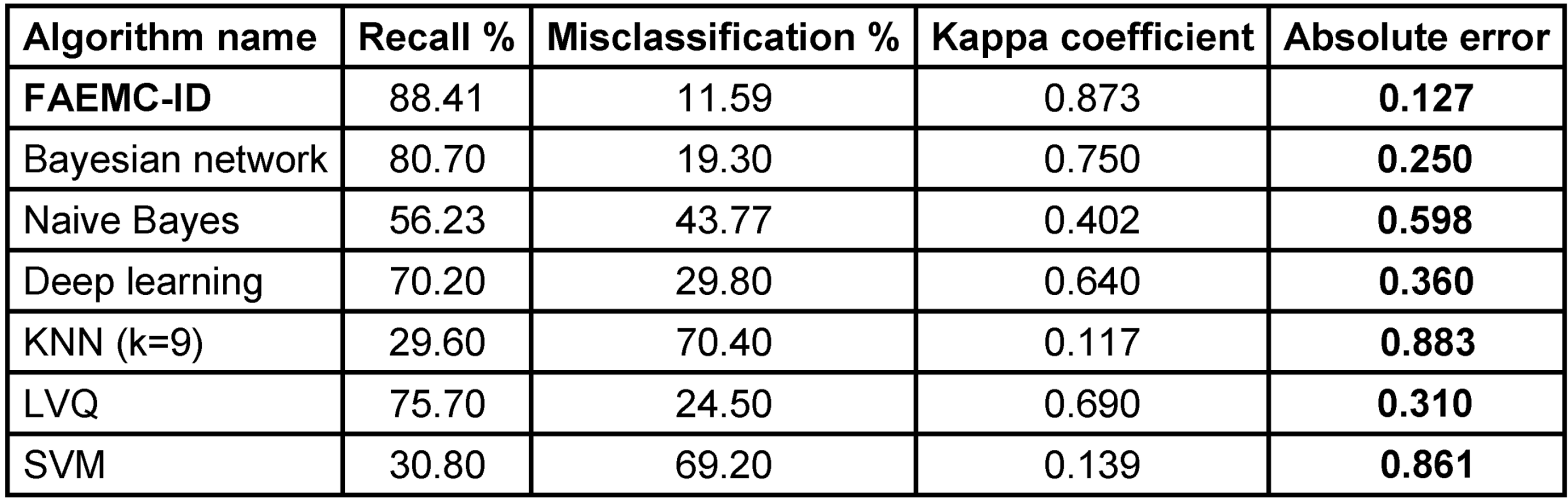
Performance of the proposed algorithms in comparison with other algorithms
